# Factors associated with diagnostic and treatment intervals in colorectal cancer: A linked data study

**DOI:** 10.1002/ijc.35414

**Published:** 2025-03-13

**Authors:** Allison Drosdowsky, Karen E. Lamb, Luc te Marvelde, Peter Gibbs, Catherine Dunn, Ian Faragher, Ian Jones, Maarten J. IJzerman, Jon D. Emery

**Affiliations:** ^1^ Department of General Practice and Centre for Cancer Research The University of Melbourne Parkville Australia; ^2^ Melbourne School of Population and Global Health The University of Melbourne Parkville Australia; ^3^ Victorian Cancer Registry Melbourne Australia; ^4^ The Walter and Eliza Hall Institute of Medical Research Melbourne Australia; ^5^ Western Health Melbourne Australia; ^6^ Department of Surgery University of Melbourne Parkville Australia; ^7^ Primary Care Collaborative Cancer Clinical Trials Group (PC4) Carlton Australia

**Keywords:** cancer, diagnostic intervals, early diagnosis, health system policy, treatment intervals

## Abstract

This research aimed to assess the length of intervals before diagnosis and treatment for colorectal cancer in Australia using linked datasets, and to determine any factors associated with interval length. A colorectal cancer clinical registry was linked to general practice electronic medical record data and routinely collected hospital referral datasets to determine the length of four key intervals in the time before first treatment. Cox proportional hazards regression was used to assess associations between individual characteristics (sociodemographic variables such as age and sex, and disease characteristics such as cancer subtype and treatment approach) and the length of each interval. Sample sizes available for analysis varied by interval, ranging from 99 to 9359. The median interval length ranged from 21 (IQR 5–38) days for the time between diagnosis and treatment to 63 (IQR 24–218) days for the time between first presentation and diagnosis. Overall, few measured characteristics were associated with the lengths of any of the intervals. Of note, shorter diagnostic intervals were associated with presenting to the general practitioner with alarm symptoms, and people proceeding to surgery as initial treatment had shorter times to treatment than any other treatment modality. Given disease and medical system factors were associated with interval length, broad improvements to the overall efficient functioning of the healthcare system are likely to improve timeliness. More targeted interventions could focus on processes at the transitions between different levels of the healthcare system and implementing recommended maximum lengths of intervals along the diagnostic and treatment pathway.

## Graphical abstract

What's new?

Colorectal cancer (CRC) is often diagnosed not by screening but because symptoms occur. Previous studies investigating the length of time between presentation and diagnosis have focused on patient characteristics, but here, the authors also look at how provider and health system factors contribute to longer diagnostic intervals, using an Australian dataset. They found a few key areas that could be improved, such as improving transitions between different components of healthcare and better identification of patients who present with non‐alarm symptoms.

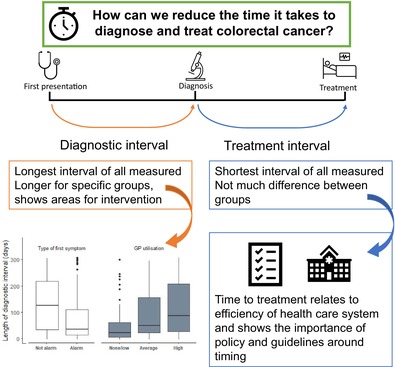

**Abbreviations**
ACCORD: Australian Comprehensive Cancer Outcomes and Research Database
EMR: Electronic Medical Record
ESAP: Elective Surgery Access Policy
GP: General Practitioner
HR: Hazard Ratio
IQR: Interquartile Range
NPS: National Prescribing Service
TWW: Two Week Wait
VINAH: Victorian Integrated Non‐Admitted Health Dataset

## 1 INTRODUCTION

Colorectal cancer is an increasing cause of cancer‐related morbidity and mortality worldwide. In Australia, it is the fourth most diagnosed cancer and the second most common cancer cause of death, with incidence expected to rise as the population ages.^1^ Despite the existence of a National Bowel Cancer Screening Program, limited uptake means many colorectal cancers are diagnosed symptomatically through contact with the healthcare system.^2^ Ensuring the expedient and efficient diagnosis and management of colorectal cancer is therefore a priority. One of the common targets outlined by cancer control strategies to improve this is to reduce the time it takes to diagnose and treat cancer. There is growing evidence that for most intervals, there is an association between longer times and poorer outcomes, although this relationship may not be straightforward or linear.^3^ In addition, timeliness is often used as a marker of quality care, reflecting healthcare efficiency and patient‐centredness and therefore represents a key component of quality benchmarking.

We need to understand the key factors associated with the length of diagnostic and treatment intervals to inform health system change. Previous research has indicated that there are some individual patient and provider characteristics associated with interval length. For example, continuity of care and more serious symptoms, such as bowel obstruction, are both associated with shorter times to diagnosis.^4^, ^5^ In addition, how the healthcare system itself is designed (e.g., whether general practitioners have a ‘gatekeeping role’ for onward referrals) can affect the time to diagnosis^6^ as well as cancer outcomes.^7^ Studies from Australia have largely focused on the difference in times between rural and urban patients^8^ and on post‐diagnosis time intervals.^9^ This exploratory study aimed to examine associations between a broader range of individual and medical provider characteristics and the length of specific intervals before diagnosis and treatment of colorectal cancer.

## 2 METHODS

### 2.1 Study design and setting

A retrospective linked data study utilizing patient‐level data from across the healthcare system. The data comes from participating healthcare services in the state of Victoria, Australia. Australia has a universal, publicly funded healthcare system. Primary care delivered by General Practitioners (GPs) is the first point of contact for many people eventually diagnosed with cancer, and GPs have a gatekeeper role in referring people on to specialist care.

### 2.2 Data sources

Details of the linked data used in this study are described elsewhere.^10^ In brief, patients from a colorectal cancer clinical registry (ACCORD; Australian Comprehensive Cancer Outcomes and Research Database^11^) from three public metropolitan hospitals were linked to two sources of general practice electronic medical record (EMR) data (MedicineInsight,^12^ a national general practice data program developed by NPS MedicineWise and now managed by the Australian Commission on Safety and Quality in Health Care, and Patron^13^), and hospital administrative data (VINAH; Victorian Integrated Non‐Admitted Health dataset^14^).

ACCORD contains demographic and clinical information, including details regarding diagnosis and treatment, on consecutive patients seen at the health service. Because it contains definitive information on colorectal cancer diagnosis, ACCORD was used as the base dataset to identify patients for inclusion in the cohort. This study had information on patients diagnosed from 1987 to 2021.

ACCORD patients were linked to two general practice datasets that contain information on episodes of care, including reasons for presenting to primary care and investigations ordered by GPs. Patron data comes from 123 Victorian practices for the years 1997 to 2020, and the Victorian cohort of MedicineInsight comes from 145 practices for the years 2007 to 2017.

Finally, information on referrals into the hospital system was taken from VINAH, which includes information about dates of referral and scheduled appointments. This study had access to VINAH from two of the participating hospitals from 2013 to 2020.

### 2.3 Linkage and data processing

The data were linked on an individual, probabilistic basis by BioGrid Australia using Grhanite, a privacy‐protecting technology that was developed for use with general practice medical records.^15^ Grhanite allows for matching with a low level of error while ensuring deidentification of sensitive records. Study populations were derived by investigators from the full dataset populations specifically for this study.

### 2.4 Participants

Eligible patients for this study were aged over 18 years with a confirmed diagnosis of colorectal cancer recorded in the ACCORD registry. The sample size for this study was pragmatic; each univariable regression included all patients with sufficient data to calculate the diagnostic or treatment interval and data on the characteristic of interest.

### 2.5 Outcomes

**FIGURE 1:**
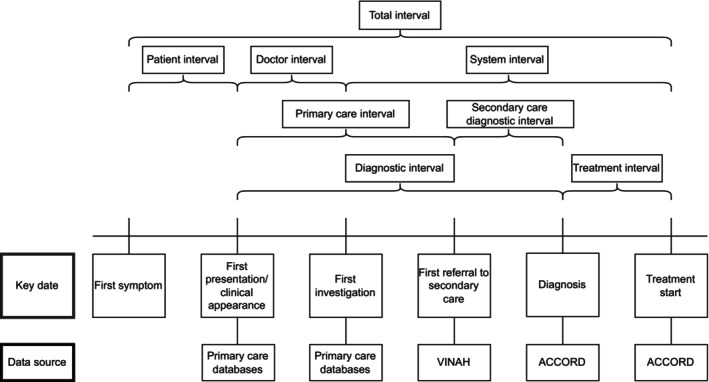
Interval definitions used in this study.

The intervals studied (see Figure 1) were based on the framework of intervals outlined in the Aarhus statement.^16^ Of the eight intervals, four could be calculated from the linked data. These were the diagnostic interval, doctor interval, secondary care diagnostic interval, and treatment interval. The dates and data sources required to determine each interval are shown in Figure 1. Date of diagnosis and first treatment were recorded in ACCORD, and date of referral to hospital in VINAH. Date of first presentation in general practice and date of first investigation were taken from the general practice EMR datasets. Date of first presentation was defined as the first relevant encounter in the year before diagnosis with a symptom that could be attributed to colorectal cancer, based on clinical practice guidelines.^17^ Date of first investigation was defined as the earliest relevant test (blood tests, faecal occult blood tests and imaging) after first presentation but before diagnosis.

### 2.6 Sociodemographic and disease‐related characteristics

Pre‐existing frameworks of factors associated with access to care were used to identify characteristics that could be associated with interval length.^18^ Among these, characteristics with data available within the linked datasets that had minimal missing data were considered. Not all characteristics were deemed relevant for all intervals; for example, the type of first treatment was only relevant for the treatment interval. Age, sex, area‐level socioeconomic disadvantage, and remoteness based on the patients' residential postcode at the time of diagnosis, and type of cancer (colon or rectal) were considered for all four intervals. Full details on the characteristics are provided as Table 1.

**TABLE 1:** Variables of interest.

Characteristic	Source	Description	Interval
Sex	ACCORD	Male or female	All
Age at diagnosis	ACCORD	Measured in years	All
Socioeconomic status	ACCORD	Quintiles 1 (most disadvantaged) to 5 (least disadvantaged); measured at area‐level using patient's residential postcode converted to quintiles of the Index of Relative Socio‐economic Deprivation	All
Remoteness of residence	ACCORD	Three categories Major City, Inner Regional, Outer Regional/Remote; collapsed from the five categories of the Australian Statistical Geography Standard Remoteness Structure 2016 [ref ARIA+] (Major City, Inner Regional, Outer Regional, Remote and Very Remote). Based on the postcode of the patient's residential address^a^	Diagnostic, treatment
Type of cancer	ACCORD	Colon or rectal	All
Type of treatment	ACCORD	Surgery, chemotherapy, radiotherapy or chemoradiotherapy; earliest recorded treatment	Treatment
Presenting symptoms	Primary care datasets	Alarm or not alarm; from stated reason for first encounter, alarm when reason for encounter clearly states reason related to colorectal cancer, and not alarm when description is less certain (e.g., ‘diarrhoea’ vs. ‘GI symptoms’)	Diagnostic, doctor
GP attendances	Primary care datasets	0–7 attendances (none/low), 8–22 attendances (average), >22 attendances (high) in a 2‐year period before diagnosis; categories modified from reports of Australian population general practice attendance	Diagnostic, doctor
Comorbidity	Primary care datasets	Yes or no; where yes is the presence of any of the following comorbidities: irritable bowel syndrome, inflammatory bowel disease, coeliac disease, diverticular disease, endometriosis, recorded in the general practice medical record before diagnosis	Diagnostic, doctor

^a^ No patients lived in ‘Very Remote’ areas.

### 2.7 Statistical methods

Sample characteristics and interval length were summarised using descriptive statistics and compared, where possible, to characteristics of all colorectal cancer patients from Victoria from 2022.^19^, ^20^ Univariable Cox proportional hazards regression models were used to assess associations between each individual factor and each interval length. The proportional hazards assumptions for each individual factor were assessed by using Schoenfeld residuals. Visual assessment showed all factors met this assumption. Some observations for the treatment interval had the same start and end date. When this occurred (18% of cases), a value of 0.5 days was used to avoid interval lengths of zero. A sensitivity analysis was performed with these cases removed, which had no effect on the reported associations. Because all participants had complete data for the length of the specific interval, there was no censoring. The resulting hazard ratio (HR) can be interpreted as the multiplicative difference in interval lengths between levels of the factor for categorical variables and per unit increase for continuous variables. For example, an HR of two means that the non‐referent group had an interval half that of the referent group.^21^ Analyses were performed in R using the survival, ggplot, and ggpubr packages.

## 3 RESULTS

**FIGURE 2:**
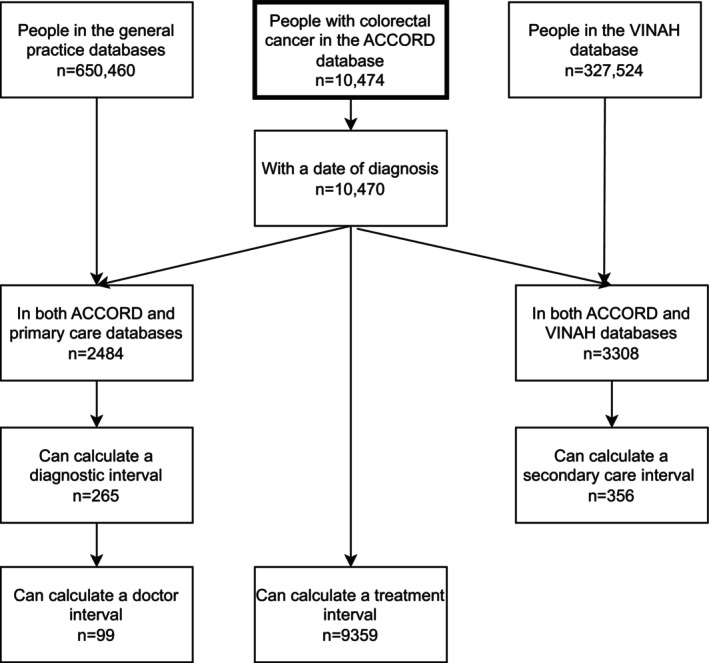
Flowchart of sample selection.

Sample characteristics are described in Table 2, alongside descriptive statistics for colorectal cancer patients across the whole state of Victoria. Sample sizes ranged from 99 for the doctor interval to 9359 for the treatment interval (see Figure 2). Characteristics across samples were similar; the mean age ranged from 66.0 (SD: 14.4) to 70.1 (12.1) years, slightly over half of the patients were male (56.5%–62.7%), most had colon cancer (69.1%–71.9%) and were from major cities of Victoria (90.3%–97.0%). The characteristics of patients (age, sex, cancer type and stage) in the samples in this study were broadly similar to the wider population of Victorians with colorectal cancer. However, there was a much higher proportion of patients from metropolitan cities in the study samples (range 90%–97% in the study samples compared to 69% in Victorian population) due to the health services participating in the study all being located in a metropolitan city.

**TABLE 2:** Description of each interval sample.

Interval	Diagnostic	Doctor	Secondary care diagnostic	Treatment	Victorians with colorectal cancer
	*n* = 265	*n* = 99	*n* = 434	*n* = 9359	
Interval length (days)					
Median (IQR)	63 (24, 218)	43 (14.5, 143)	27 (3, 111.8)	21 (5, 38)	
Range	1, 365	1, 280	0, 365	0, 1213	
Age					
Mean (SD)	66.0 (14.4)	70.1 (12.1)	67.3 (12.4)	66.8 (13.1)	70
Range	24, 93	33, 93	26, 91	18, 101	
Sex					
Male	154 (58.1)	58 (58.6)	272 (62.7)	5290 (56.5)	52.0%
Female	111 (41.9)	41 (41.4)	162 (37.3)	4069 (43.5)	48.0%
SES					
1 Most disadvantaged	63 (23.8)	24 (24.2)	161 (37.1)	1733 (18.5)	23.1%
2	57 (21.5)	24 (24.2)	77 (17.7)	1247 (13.3)	20.7%
3	61 (23.0)	18 (18.2)	114 (26.3)	1811 (19.4)	19.3%
4	43 (16.2)	19 (19.2)	35 (8.1)	2455 (26.2)	19.5%
5 Least disadvantaged	35 (13.2)	10 (10.1)	39 (9.0)	1833 (19.6)	17.4%
Missing	6 (2.3)	4 (4.0)	8 (1.8)	280 (3.0)	
Remoteness					
Major city	242 (91.3)	90 (90.9)	421 (97.0)	8454 (90.3)	69.0%
Inner regional	17 (6.4)	5 (5.1)	6 (1.4)	528 (5.6)	31.0%^a^
Outer Regional/remote	0 (0)	0 (0)	0 (0)	99 (1.1)	
Missing	6 (2.3)	4 (4.0)	7 (1.6)	278 (3.0)	
Type of cancer					
Colon	183 (69.1)	71 (71.7)	312 (71.9)	6621 (70.7)	70.0%
Rectal	82 (30.9)	28 (28.3)	122 (28.1)	2738 (29.3)	30.0%
ACPS stage					
A	49 (18.5)	19 (19.2)	94 (21.7)	1715 (18.3)	21%^b^
B	71 (26.8)	26 (26.3)	103 (23.7)	2921 (31.2)	21%
C	59 (22.3)	24 (24.2)	92 (21.2)	2669 (28.5)	19%
D	56 (21.1)	18 (18.2)	69 (15.9)	1345 (14.4)	19%
Missing	30 (11.3)	12 (12.1)	76 (17.5)	709 (7.6)	21%
Year of diagnosis					
1980–1989	0 (0)	0 (0)	0 (0)	47 (0.5)	
1990–1999	0 (0)	0 (0)	0 (0)	552 (5.9)	
2000–2009	39 (14.7)	9 (9.1)	13 (3.0)	2953 (31.6)	
2010–2019	219 (82.6)	87 (87.9)	398 (91.7)	5189 (55.4)	
2020–2022	7 (2.6)	3 (3.0)	23 (5.3)	618 (6.6)	
Type of first treatment					
Surgery	178 (67.2)	70 (70.7)	288 (66.4)	8025 (85.7)	
ChemoRT	43 (16.2)	12 (12.1)	54 (12.4)	1060 (11.3)	
Chemotherapy	7 (2.6)	3 (3.0)	10 (2.3)	170 (1.8)	
Radiotherapy	2 (0.8)	1 (1.0)	5 (1.2)	104 (1.1)	
None	35 (13.2)	13 (13.1)	77 (17.7)	0 (0)	
History of cancer					
No	25 (9.4)	5 (5.1)	10 (2.3)	1692 (18.1)	
Yes	4 (1.5)	2 (2.0)	3 (0.7)	287 (3.1)	
Not recorded	236 (89.1)	92 (92.9)	343 (97.0)	7380 (78.9)	
Type of first symptom					
Alarm	167 (63.0)	53 (53.5)			
Not‐alarm	98 (37.0)	46 (46.5)			
GP attendances in the 2 years before diagnosis					
None/low (<8)	54 (20.4)	6 (6.1)			
Average (8–22)	97 (36.6)	28 (28.3)			
Frequent (>22)	114 (43.0)	65 (65.7)			
Comorbidity recorded					
No	247 (93.2)	88 (88.9)			
Yes	18 (6.8)	11 (11.1)			

^a^ Regional and remote areas combined.

^b^ Reported as stage I–IV.

On average, the diagnostic interval was the longest interval (see Table 2), with the greatest variability (median 63 days, interquartile range [IQR] 24–218). The treatment interval was the shortest, with the least variation (median 21 days, IQR 5–38).

Interval length by each characteristic of interest and regression results are shown in Table 3 and in Figures S1 (diagnostic and doctor intervals) and S2 (secondary care diagnostic and treatment intervals). Few characteristics were found to be associated with the lengths of any interval. Of note, two features of general practice use were associated with the length of the diagnostic interval. Firstly, patients who reported to general practice with alarm symptoms of colorectal cancer had shorter diagnostic intervals (median 26, IQR 20–133 days) than those who presented with non‐alarm symptoms (median 153, IQR 45–260 days). Modelling results supported this finding (HR 1.56, 95% CI 1.21–2.01). Second, patients who frequently attended general practice in the 2 years preceding diagnosis were diagnosed more slowly than patients who visited the GP less frequently. The median length of the interval increased from 30 days (IQR 11–77) for people who attended less than eight times to 107 (IQR 36–248) days for more frequent attenders (>22 times). This is also shown in the modelling results (8–22 vs. <8 times: HR 0.67, 95% CI 0.48–0.94; 22+ vs. <8 times: HR 0.52, 95% CI 0.38–0.72).

**TABLE 3:** Regression results.

	Diagnostic interval		Doctor interval		Secondary care diagnostic interval		Treatment interval	
	Median (IQR)	HR (95%CI)	Median (IQR)	HR (95%CI)	Median (IQR)	HR (95%CI)	Median (IQR)	HR (95%CI)
Sex								
Female	71 (24, 224)	ref	73 (23, 176)	ref	31.5 (3, 105.8)	ref	20 (4, 36)	ref
Male	54 (25, 197)	1.04 (0.81, 1.33)	39 (14, 110)	1.02 (0.68, 1.53)	23.5 (4, 114)	1.04 (0.86, 1.27)	22 (6, 39)	**0.94 (0.90, 0.97)**
Age								
Mean centred		1.00 (0.99, 1.01)		1.01 (0.99, 1.02)		1.00 (0.99, 1.002)	–	1.02 (0.99, 1.04)
SES								
1 Most disadvantaged	76 (32.5, 223.5)	0.89 (0.62, 1.28)	38 (15, 98)	1.40 (0.73, 2.68)	24 (2, 105)	1.02 (0.80, 1.29)	21 (1, 38)	1.02 (0.95, 1.09)
2	71 (19, 238)	0.90 (0.62, 1.30)	55 (8, 140)	1.56 (0.82, 2.96)	34 (4, 102)	1.02 (0.76, 1.36)	20 (2, 37)	1.05 (0.98, 1.13)
3	41 (28, 125)	ref	76 (34, 173)	ref	15.5 (2.3, 101.8)	ref	21 (5, 38)	ref
4	92 (34.5, 226.5)	0.74 (0.50, 1.10)	67 (30, 105)	1.01 (0.52, 1.96)	42 (5, 182)	0.82 (0.56, 1.20)	22 (7, 39)	0.98 (0.93, 1.05)
5 Least disadvantaged	52 (17.5, 105)	1.05 (0.69, 1.60)	33 (13, 217)	1.21 (0.55, 2.67)	30 (9.5, 109)	1.01 (0.70, 1.46)	18 (7, 34)	**1.08 (1.01, 1.16)**
Remoteness								
Major city	62 (23, 201)	ref					21 (5, 37)	ref
Inner regional	83 (45, 229)	0.86 (0.52, 1.41)					23 (8, 43)	**0.84 (0.77, 0.92)**
Outer regional/remote	–	–					14 (4, 31)	1.07 (0.88, 1.31)
Type of cancer								
Colon	68 (24, 225)	ref	67 (21, 174)	ref	28 (3, 134.8)	ref	16 (2, 33)	ref
Rectal	60 (28, 180.5)	1.17 (0.90, 1.52)	32 (14, 69)	**1.61 (1.03, 2.52)**	20 (4.3, 75.5)	**1.29 (1.04, 1.60)**	29 (16, 47)	**0.66 (0.63, 0.69)**
Type of first treatment								
Surgery							18 (2, 35)	ref
ChemoRT							32 (23, 43)	**0.74 (0.69, 0.79)**
Chemotherapy							27 (15, 41)	**0.85 (0.73, 0.99)**
Radiotherapy							32 (20.5, 47)	**0.72 (0.59, 0.88)**
Type of first symptom								
Alarm	26 (20, 133)	**1.56 (1.21, 2.01)**	78 (24, 174)	1.39 (0.93, 2.01)				
Not‐alarm	153 (45, 260)	ref	37 (13, 88)	ref				
GP attendances in the 2 years before diagnosis								
None/low (<8)	30 (11, 77)	ref	32 (21, 163)	ref				
Average (8–22)	62 (30, 188)	**0.67 (0.48, 0.94)**	69 (27, 149)	2.18 (0.88, 5.4)				
Frequent (>22)	107 (36, 248)	**0.52 (0.38, 0.72)**	39 (14, 130)	1.57 (0.67, 3.7)				
Comorbidity								
No	61 (23, 204)	ref	45 (17, 141)	ref				
Yes	172 (38, 278)	0.70 (0.43, 1.13)	39 (9, 156)	0.92 (0.49, 1.73)				

*Note*: intervals measured in days. Bold indicates statistical significance where *p* < 0.05.

The length of the treatment interval was found to differ for the different types of treatment (see Table 3). On average, surgery had the shortest treatment interval (median 18, IQR 2–35 days) compared to 27 days (IQR 15–41) for chemotherapy, 32 days (IQR 20.5–47) for radiotherapy, and 32 days (IQR 23–43) for chemoradiotherapy. In addition, the treatment interval differed by cancer type. The median treatment interval was shorter for colon cancer patients (median 16, IQR 2–33 days) than for rectal cancer patients (median 29, IQR 16–47 days), with an estimated HR of 0.66 (95% CI 0.63–0.69) for rectal compared to colon cancer patients. Cancer type was also associated with the length of the doctor interval, with rectal cancer patients taking less time to first investigation after presentation in general practice (median 32, IQR 14–69 days for rectal cancer versus median 67, IQR 21–174 days for colon cancer, HR for rectal cancer 1.61, 95% CI 1.03–2.52).

## 4 DISCUSSION

This study determined the lengths of four key intervals in the time before diagnosis and treatment in colorectal cancer in a cohort of Australians with colorectal cancer and examined whether any sample characteristics were associated with interval length. Although few characteristics were associated with longer intervals, there were some notable findings.

Two characteristics were associated with longer diagnostic intervals: having non‐alarm symptoms and frequent attendance in general practice in the 2 years preceding diagnosis. Notably, none of the measured individual sociodemographic characteristics (including age, sex, remoteness, and socioeconomic status) were found to be associated with pre‐diagnosis interval lengths. Although, in contrast to the studies reported in the introduction, this finding is consistent with some previous research.^5^ The finding that patients with non‐alarm symptoms of colorectal cancer spend longer in the diagnostic period is consistent with previous research.^5^, ^21^, ^22^ Colorectal cancer can present with a ‘broad’ symptom profile, with symptoms showing varying predictive capacity and some notable alarm symptoms.^23^ These symptoms are also fairly common within the general community. Similarly, our finding that patients who are more frequent users of general practice had longer diagnostic intervals has also been seen in previous research.^4^, ^5^ This may reflect that patients who attend frequently have more complex health conditions, although we found no association between specific comorbidities and the length of pre‐diagnostic intervals. There were, however, relatively few patients in our cohort with the pre‐specified comorbidities. That none of these factors were also associated with the doctor interval, which is a subset of the diagnostic interval, is an area for further study, as the smaller sample size of the former in the study may have precluded the power to assess these differences.

The pre‐diagnostic intervals were the longest overall of the intervals under study. This period includes time spent in both primary and secondary care, and the transition between the two. Referrals and transitions between different levels of care are a necessary part of the diagnostic journey for people with cancer but can be a major contributor to the overall total diagnostic interval. For example, the gatekeeper role of GPs referring on to specialist care may be associated with longer diagnostic intervals, and the lack of systems to enable care coordination across levels of the system, such as unified medical records, or lack of care navigators in the Australian context may also increase delays.^6^

A key pre‐diagnostic interval is the secondary care diagnostic interval, between being referred to a specialist and diagnosis. In colorectal cancer, in the Australian context, this is likely the referral for a diagnostic colonoscopy, which is often preceded by assessment by a specialist gastroenterologist or surgeon. The time period spent between referral and appointment with a specialist has been referred to as the ‘hidden waitlist’ in the Australian context, because waiting times for outpatient appointments are covered by a policy that garners less public attention than surgical waitlists but that allows for specialist appointments to occur up to 1‐year post referral in routine cases and 30 days in high‐priority cases.^24^ In addition, this policy does not cover time to colonoscopy itself. In this study, the average time between both first presentation in primary care or referral to secondary care and diagnosis, which was likely a colonoscopy, was 117 and 73 days, respectively, well in excess of guidelines that recommend a maximum of 30 days between presentation and colonoscopy,^25^ although evidence also suggests 120 days as a maximum.^17^

The factors associated with the length of the pre‐diagnostic period and the length of the intervals themselves indicate several areas that could benefit from targeted improvement. Given the longer intervals, in this study, for people who presented more frequently to their GP or with non‐alarm symptoms, interventions in the primary care setting that may improve timeliness are those that assist with reducing the uncertainty around pathways, for example, by increasing the adherence to and utilisation of clinical practice guidelines. Interventions that utilise routinely captured data and electronic decision support systems can monitor large amounts of data about symptoms or investigations over time without increasing the burden on GPs and might provide an opportunity to reduce the diagnostic interval.

However, evidence suggests that the subsequent stages of the diagnostic pathway, during diagnostic investigations, may be the largest source of avoidable delays.^26^ To improve the appropriate use of colonoscopy, guidelines delineating for whom colonoscopy is recommended and appropriate prioritisation of referrals have both been suggested as methods that could improve access in Australia. There is evidence that colonoscopy may be used incorrectly as a primary screening measure for people at average risk of cancer.^27^ The burden of colonoscopies specifically on the publicly funded healthcare system can be mitigated by utilising public‐private partnerships.^28^ The ability of the healthcare system to provide more colonoscopies is largely reliant on the workforce, with this considered a key risk to the ability to meet future demand for services.^29^ While most colonoscopies are performed by non‐GP specialists in Australia, capacity has been increased by broadening the types of clinicians that are able to perform colonoscopies, such as nurse endoscopists and GPs, usually in rural and remote settings.^30^ Strategies such as direct access to colonoscopy, removing the requirement for a specialist appointment or replacing it with nurse‐led clinics, have also shown, in specific programs, increased capacity for, and reduced time to, colonoscopy.^31^

Outside of colonoscopy, targeted diagnostic approaches including diagnostic centres and guidelines for triaging and referring have worked in other countries to ensure appropriate use of resources, reduced times to diagnosis, and improved outcomes.^32^

Another strategy that could be used in the pre‐diagnostic period is the implementation of cancer‐specific timeliness policies, given their current absence in Australia. The UK has used such approaches since 2000, most notably the Two Week Wait (TWW) program where urgent referrals with a suspicion of cancer should be seen by a specialist within 2 weeks. The use of the TWW program has shown benefits, including a reduction in mortality.^33^ Lessons could be taken from the current TWW reform, which has highlighted the issues surrounding using timeliness as a benchmark. There is evidence to suggest the TWW program pressured services to provide care quickly but in ways that may not have improved the quality of care provided.^34^ Newer standards will replace the endpoint of the targeted time with diagnosis rather than a specialist appointment. In addition, implementing time‐based policies is also limited by the lack of evidence surrounding what constitutes a detrimental length of time for many intervals, as well as difficulty in determining what would constitute the start time of a pre‐diagnostic interval, given the uncertainty around colorectal cancer symptoms.

In contrast to the pre‐diagnostic intervals, the treatment interval was shortest overall of all the intervals studied, with the least variation, and was significantly shorter when surgery was the treatment. This is also reflected in our finding that colon cancer, compared to rectal cancer, was associated with shorter times to treatment, as surgery is the primary treatment type for this cancer. In Australia, time to surgery is managed through the Elective Surgery Access Policy (ESAP) policy, which states that patients with cancer should have surgery within 30 days. Hospitals failing to meet these times are subject to a range of interventions and additional monitoring. There are timeliness recommendations for other kinds of cancer treatment, but they are not subject to such stringent monitoring, so they may have lower adherence. Patients treated with all other modalities had longer treatment intervals than those who had surgery. This may reflect greater time for treatment planning but potentially also relate to issues of access.

The relative shortness of the treatment interval may also reflect the different factors that might be causing delays. Once a patient is diagnosed, there is less uncertainty around appropriate pathways, and concern around timeliness is driven by logistical and practical issues, such as scheduling surgery. Indeed, even before the ESAP was enacted, evidence suggests time to surgery was short and reducing.^35^

Focusing on timeliness can provide improvements in outcomes and allows for monitoring and benchmarking of the diagnostic and treatment pathway. However, time itself is a surrogate measure for the myriad components of the healthcare system that are involved with people waiting for cancer diagnosis and treatment. At a broad level, any intervention that strengthens the healthcare system as a whole is likely to help reduce times to treatment for cancer patients. Many of these broader interventions have the added benefit of improving the whole healthcare system, beyond solely improving the outcomes for people with colorectal cancer.

## 5 LIMITATIONS

While the use of linked data is a strength of this study, enabling patients to be followed through the entire diagnostic process, this results in several limitations. The chief limitation is the lack of population‐based data and data on potentially important characteristics. Both ACCORD and VINAH come from metropolitan hospitals, which limited the representation of patients in the broader population, with an under‐representation of regional populations. This could explain the lack of association between remoteness and interval length. Furthermore, due to the difficulty in accessing data, this study only had access to VINAH data from two of the three ACCORD hospitals and had no population‐based emergency presentation data. This is a limitation because it is estimated that approximately 10% of colorectal cancer patients present through the emergency department.^36^ Additionally, VINAH does not contain information on reasons for referral. This made it impossible to investigate the impact of time to specialist presentation before colonoscopy, a key additional time interval of interest.

There was also great variation in the sample sizes for each of the analyses due to the use of linked data. In particular, smaller sample sizes were used in the analyses of the pre‐diagnostic intervals and intervals that had start and end dates from different data sources, such as the doctor interval. The smaller sample sizes limited the statistical power to detect differences for these intervals and were due to the fact that the different data sources had different spatial coverage across Victoria. Efforts should be made across Victoria and Australia to improve access to population‐level data sources. This would not only improve research on the treatment of cancer patients across the healthcare system but would lead to a greater understanding of patient treatment and outcomes more broadly.

Furthermore, the characteristics considered in this analysis were chosen due to availability in the routinely collected data, meaning some characteristics of potential interest could not be considered, such as disability, migrant status, language spoken at home, and Aboriginal and/or Torres Strait Islander status. Any association with socioeconomic status may also have been hidden by our use of area‐level measures, as access to individual‐level information was not available.

Finally, as this was an exploratory study, only univariable analyses were performed. It is possible some of the reported associations could be explained by other characteristics. For example, the finding that patients with rectal cancer wait longer for treatment after diagnosis could be explained by the finding that patients treated with surgery had the shortest times to treatment, and rectal cancer is commonly treated initially with non‐surgical modalities. In addition, multiple tests were performed in this study. This increases the likelihood that findings were due to chance. However, interpretation was focused on findings with clinical significance and large effects.

## 6 CONCLUSION

Ensuring cancer care is provided in a timely manner is key to limiting poorer outcomes and improving the quality of care for people with cancer. Timeliness is a common metric of quality care because it is a good indicator of a well‐functioning healthcare system. Consequently, the key to reducing the time it takes to diagnose and treat cancer may lie in strengthening the system as a whole and ensuring that all components of the system have appropriate funding and resources. This study found a few key areas that could be targeted to reduce the length of intervals in the time before and treatment for people with colorectal cancer in Australia, namely improving transitions of care between different components of the healthcare system, especially when accessing diagnostic colonoscopy, and helping with the identification of patients in primary care who present without alarm symptoms, as this subgroup had longer times to diagnosis.

## AUTHOR CONTRIBUTIONS

**Allison Drosdowsky:** Conceptualization; methodology; formal analysis; funding acquisition; visualization; writing – original draft; writing – review and editing. **Karen E. Lamb:** Conceptualization; writing – original draft; writing – review and editing; formal analysis; supervision; methodology. **Luc te Marvelde:** Conceptualization; methodology; formal analysis; writing – review and editing. **Peter Gibbs:** Data curation; resources; writing – review and editing; conceptualization; methodology. **Catherine Dunn:** Data curation; methodology; conceptualization; writing – review and editing; resources. **Ian Faragher:** Conceptualization; methodology; data curation; resources; writing – review and editing. **Ian Jones:** Conceptualization; data curation; resources; writing – review and editing; methodology. **Maarten J. IJzerman:** Conceptualization; methodology; supervision; formal analysis; writing – original draft; writing – review and editing. **Jon D. Emery:** Conceptualization; methodology; formal analysis; supervision; writing – original draft; writing – review and editing; funding acquisition.

## FUNDING INFORMATION

This project is supported by the Victorian Comprehensive Cancer Centre Strategic Research Program and the Bupa Foundation. Specific funding for investigators: JDE NHMRC Investigator grant APP1195302 and Allison Drosdowsky is supported by an NHMRC postgraduate scholarship.

## CONFLICT OF INTEREST STATEMENT

All authors declare no conflict of interests.

## ETHICS STATEMENT

This study was approved by the local ethics committee (Melbourne Health Human Research Ethics Committee, project number 202003/8). A waiver of informed consent to participate was granted based on the requirements outlined by the Australian National Health and Medical Research Council National Statement on Ethical Conduct in Human Research (2023).

## Supporting information


**DATA S1.** Supporting information.

## Data Availability

Data sources and handling of the datasets used in this study are described in the Materials and Methods. Further details and other data that support the findings of this study are available from the corresponding authors upon request.
