# Supplementary material for: Factors associated with diagnostic and treatment intervals in colorectal cancer: A linked data study
Source: Int J Cancer. 2025 Mar 13;157(4):687–97. doi: 10.1002/ijc.35414 (PMC12178107; doi:10.1002/ijc.35414)

Factors associated with diagnostic and treatment intervals in colorectal cancer: a linked data study.

Allison Drosdowsky, Karen E Lamb, Luc te Marvelde, Peter Gibbs, Catherine Dunn, Ian Faragher, Ian Jones, Maarten J IJzerman, Jon D Emery

Supplementary Figures

1. Lengths of the diagnostic and doctor intervals by characteristics of interest

2. Lengths of the secondary care diagnostic and treatment intervals by characteristics of interest

Supplementary Figure 1: Lengths of the diagnostic and doctor intervals by characteristics of interest

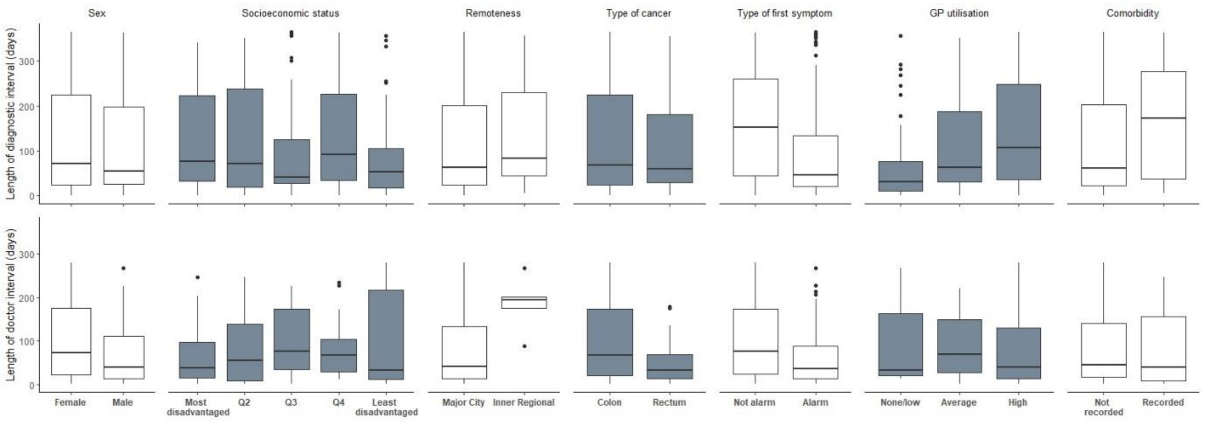

Supplementary Figure 2: Lengths of the secondary care diagnostic and treatment intervals by characteristics of interest

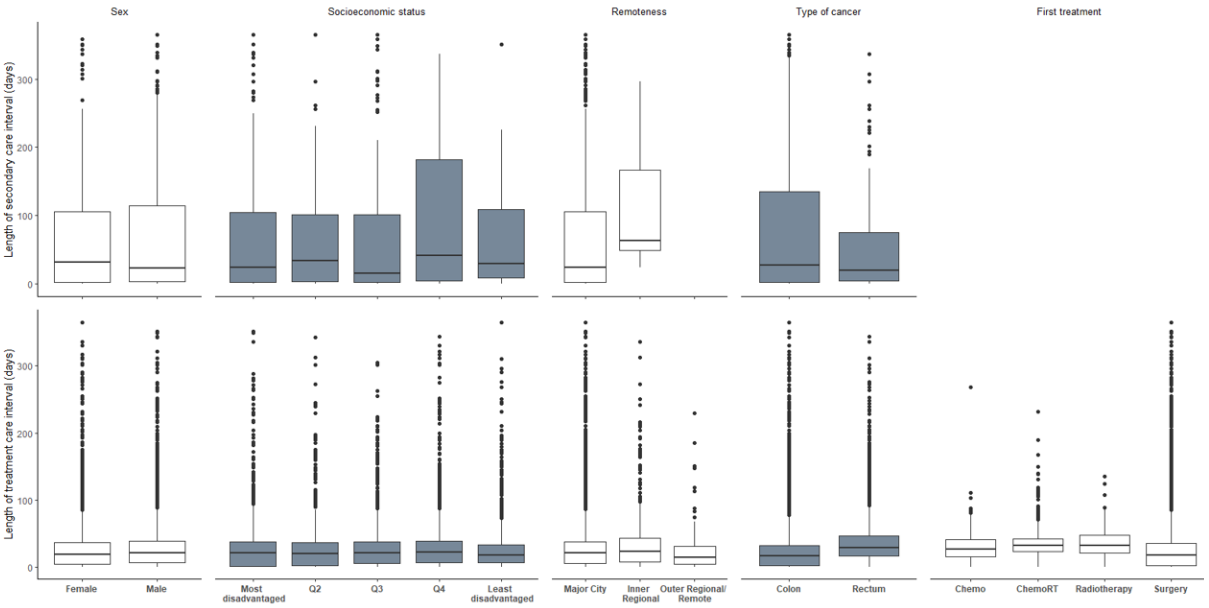

Supplement: Supplementary file 1 — DATA S1. Supporting information. [file IJC-157-687-s001.pdf]
